# Editorial bring in the new!

**DOI:** 10.1098/rsob.150264

**Published:** 2016-01-06

**Authors:** David M. Glover

The year 2015 has been a year for celebration when Royal Society publishing has marked the 350th anniversary of the first publication of its *Philosophical Transactions*, the world's first scientific journal that is still going strong (https://royalsociety.org/journals/publishing350/).

*Open Biology*, by contrast, is now an infant of a little over 4 years old but also thriving. Our aim with *Open Biology* was to establish an open access journal exploring biological systems at a cellular and molecular level of resolution, an area of biology that was underrepresented in the Society's existing journals. Above all, we wanted to have a journal run for cell and molecular biologists by practicing scientists that would bring first class articles to rapid publication without unnecessary rounds of review. We are making excellent progress towards this goal and *Open Biology* is publishing papers that are catching the attention of the community as evident from its rising impact factor (3.625 in 2012, 4.556 in 2013 and 5.784 in 2014—just one measure of a variety of journal-based metrics used by the journal to assess impact http://rsob.royalsocietypublishing.org/citation-metrics).

Now the roots are secure and we need to grow and branch out to reach more authors and readers. To do so, we rely heavily upon the ambassadorial activities of our editorial board and editors. I would like to thank all our editorial board, a particularly important body whose members actively participate in the first screening of papers and in selecting referees. Our hard-working board hail from wide geographical origins and represent all aspects of biology. As 2015 draws to a close, we are sad to say goodbye to Mark Petronczki and Dario Alessi and would like to thank them so very much for their great help in starting the journal. We are delighted to welcome Ilan Davis, Uttam Surana, Oscar Marín, Richard Wood, Ulrike Gruneberg, Ian Lipkin, Nick Tonks and Corrine Houart to join us on the board.

The final decision on publication rests with the smaller number of editors and with an increase in papers in Developmental Biology, we have begun to feel the loss of Jim Smith. Jim's wisdom and pragmatism was extremely important in guiding the journal through these early years. For some time, therefore, we have been looking for his ideal replacement and I am now delighted to announce our success in persuading Marek Mlodzik to take on this role.

Marek is Professor and Chair of the Department of Developmental and Regenerative Biology at the Icahn School of Medicine at Mount Sinai, New York. Marek's lab is well known for its studies of Wnt/Frizzled-planar cell polarity signalling and associated regulatory mechanisms using *Drosophila* as a model for *in vivo* studies, cell culture experiments and biochemistry. He is very familiar with vertebrate developmental models through his collaborations with colleagues using zebrafish or mouse models. Marek will be a superb representative for the journal among the developmental biology community and his location of the East Coast is very important to us in gaining a greater presence in the USA.

I hope you will all join me in welcoming Marek to this position. We are greatly looking forward to working with him in preparing for the challenges of 2016 and beyond. Above all, we look forward to reporting lots of successful science from a growing readership.

